# Trichotomous prognosis-based outcome analysis: an efficient and clinically relevant method for analysis of acute stroke trials

**DOI:** 10.1186/1745-6215-14-S1-O119

**Published:** 2013-11-29

**Authors:** David Barer, Eivind Berge, Else Charlotte Sandset

**Affiliations:** 1 Newcastle University, Newcastle, UK; 2 Oslo University Hospital, Oslo, Norway

## Background

Stroke trial outcomes are usually classified in ordered functional categories, e.g. the modified Rankin Scale (mRS). Using a single cut-off to distinguish "good" and "poor" outcome is statistically inefficient and could miss effects in subgroups where few patients can be expected to achieve good outcome. "Ordinal shift analysis", using ordinal logistic regression to estimate a common odds ratio for all possible outcome category thresholds, is more efficient but could still obscure clinically important differences in response among prognostic subgroups. An alternative "prognosis based outcome" (PBO) approach defines separate, clinically meaningful good and poor outcome thresholds (GOTs and POTs) within each prognostic stratum. The strata are then combined in an overall trichotomous comparison of "good"/"intermediate"/"poor" outcomes.

## Methods

We defined prognostic categories, based on initial neurological scores, and derived suitable GOTs and POTs for the mRS, using data from a hospital stroke register. We then validated these thresholds using baseline and outcome data from the Scandinavian Candesartan Acute Stroke Trial (SCAST). Finally we performed trichotomous PBO analysis on the SCAST data.

## Results

PBO analysis confirmed the overall neutral results of SCAST but did identify a notable negative treatment effect within the good prognosis group.

## Conclusions

This subgroup effect was not predicted, so requires independent confirmation. The PBO approach is clinically intuitive, can be used to analyse treatment response within different prognostic groups (as in cancer staging systems), and is statistically efficient. Also, it does not require multivariable modelling, making it easier to combine data from different studies in individual patient data meta-analysis.

